# Enhancement patterns of the normal facial nerve on three-dimensional T1W fast spin echo MRI

**DOI:** 10.1259/bjr.20201025

**Published:** 2021-01-27

**Authors:** Richard Warne, Olivia Mary Carney, George Wang, Steve Connor

**Affiliations:** 1 Department of Neuroradiology, King’s College Hospital, NHS Foundation Trust, London, UK; 2 Department of Biostatistics, University of Sydney, School of Public Health, Sydney, New South Wales, Australia; 3 School of Biomedical Engineering and Imaging Sciences, St Thomas’ Hospital, King’s College London, London, UK

## Abstract

**Objectives:**

With increasing neuroimaging applications of contemporary three-dimensional *T_1_* W fast spin echo (3D T1W FSE) sequences, it was aimed to reappraise the normal patterns of skull base facial nerve gadolinium enhancement.

**Methods:**

Pre- and post-gadolinium 3D T1W fast spin echo imaging studies (*n* = 64) were retrospectively analysed in patients without suspected facial nerve pathology. Two independent observers scored the signal at each of six skull base facial nerve segments. Wilcoxon signed-rank test was used to compare changes in signal between pre- and post-gadolinium sequences at each location, and how this differed between proprietary sequences or between the pairs of facial nerves.

**Results:**

There was significant enhancement at the fundal canalicular (16%), geniculate ganglion (96%), tympanic (45%) and mastoid (38%) facial nerve segments (*p* < 0.05). Two different proprietary sequences demonstrated similar patterns of enhancement and there was symmetry between the two sides.

**Conclusions:**

There is a differing pattern of normal facial nerve enhancement on contemporary 3D T1W FSE sequences compared to previous studies of 2D T1W SE imaging and fundal canalicular enhancement may be physiological.

**Advances in knowledge:**

This is the first study to evaluate patterns of normal facial nerve enhancement using contemporary 3D T1W FSE MRI sequences.

## Introduction

Normal patterns of skull base facial nerve gadolinium enhancement have been previously described in the context of T1W spin echo (T1W SE) and three-dimensional T1W gradient echo (3D T1W GRE) sequences.^1–6^ Contemporary 3D FSE techniques include CUBE (General Electric) and Sampling Perfection with Application optimized Contrasts using different flip angle Evolution (SPACE; Siemens). These sequences are characterized by short non-spatially selective radio-frequency pulses to significantly shorten the echo spacing and variable flip angles for the refocusing radio-frequency pulses. This suppresses blurring whilst reducing flow and chemical shift artifacts. The precise implementations vary between the different MRI equipment manufacturers, and variations in factors such as the effective echo time and flip angle may influence tissue contrast. Given that contemporary 3D T1W FSE techniques are now widely applied in neuroimaging and may be required for the evaluation of facial nerve dysfunction, it is important to re-appraise the normal variation in facial nerve enhancement when using these sequences, such that physiological post-gadolinium imaging appearances are not misinterpreted as pathological.

Our objectives were to evaluate normal patterns of skull base facial nerve gadolinium enhancement using contemporary 3D T1W FSE sequences, to determine whether there was a similar pattern of gadolinium enhancement when analysing specific 3D T1W FSE proprietary (CUBE and SPACE) sequences and to investigate whether physiological skull base 3D T1W FSE facial nerve gadolinium enhancement was symmetric.

## Methods and materials

The study was approved by the local institutional review board as a service evaluation. Retrospective analysis of (*n* = 64) obtained in a 2-year period from 2015 to 2017 for patients undergoing imaging following tertiary referral for headache investigation. This protocol was chosen since it included pre- and post-gadolinium 3D T1W FSE sequences of the brain and skull base. A priori exclusion criteria were any leptomeningeal or neural MRI abnormality, previous intracranial or petrous bone surgery, neurological signs, history of facial nerve or other cranial nerve palsy and artifactual degradation.

### Imaging protocols

Imaging was performed on either Magnetom Aera (Siemens, Erlangen, Germany) or Signa (GE, Milwaukee,WI) 1.5T systems with a 64-channel head/neck coil. Either 3D T1W SPACE (Siemens) or CUBE (GE) sequences were performed both pre-contrast (without fat saturation) and then immediately after administration of an i.v. single bolus of gadobutrol (Gd-DO3A-butrol,Gadovist^®^, Bayer Shering Pharma AG, Berlin, Germany) at a dose of 0.1 mmol/kg (1 mmol ml^−1^). The MRI parameters for the 3D SPACE sequence were Repetition Time (TR) 600 ms (ms) /Time to Echo (TE) 7.2 ms/flip angle (FA) variable/ voxel size 1 × 1 × 1 mm/ matrix 256 × 256/field of view 256 mm/ bandwidth 630 Hz/Px/ echo train length 24/acquisition time 6 min 4 s and parameters for the 3D CUBE T1W sequence were TR 600 ms/ TE 24 ms/ FA 90 deg/ voxel size 1 × 1 × 1 mm/ matrix 256/field of view 256 mm/ bandwidth 31.1 Hz/Px/ echo train length 36/acquisition time 4 min 56 s. Key disparities between the sequences include differing echo train length and consequently effective echo times, as well as differing approaches to the flip angles (variable with SPACE and fixed with CUBE).

### Imaging analysis

**Figure 1.**
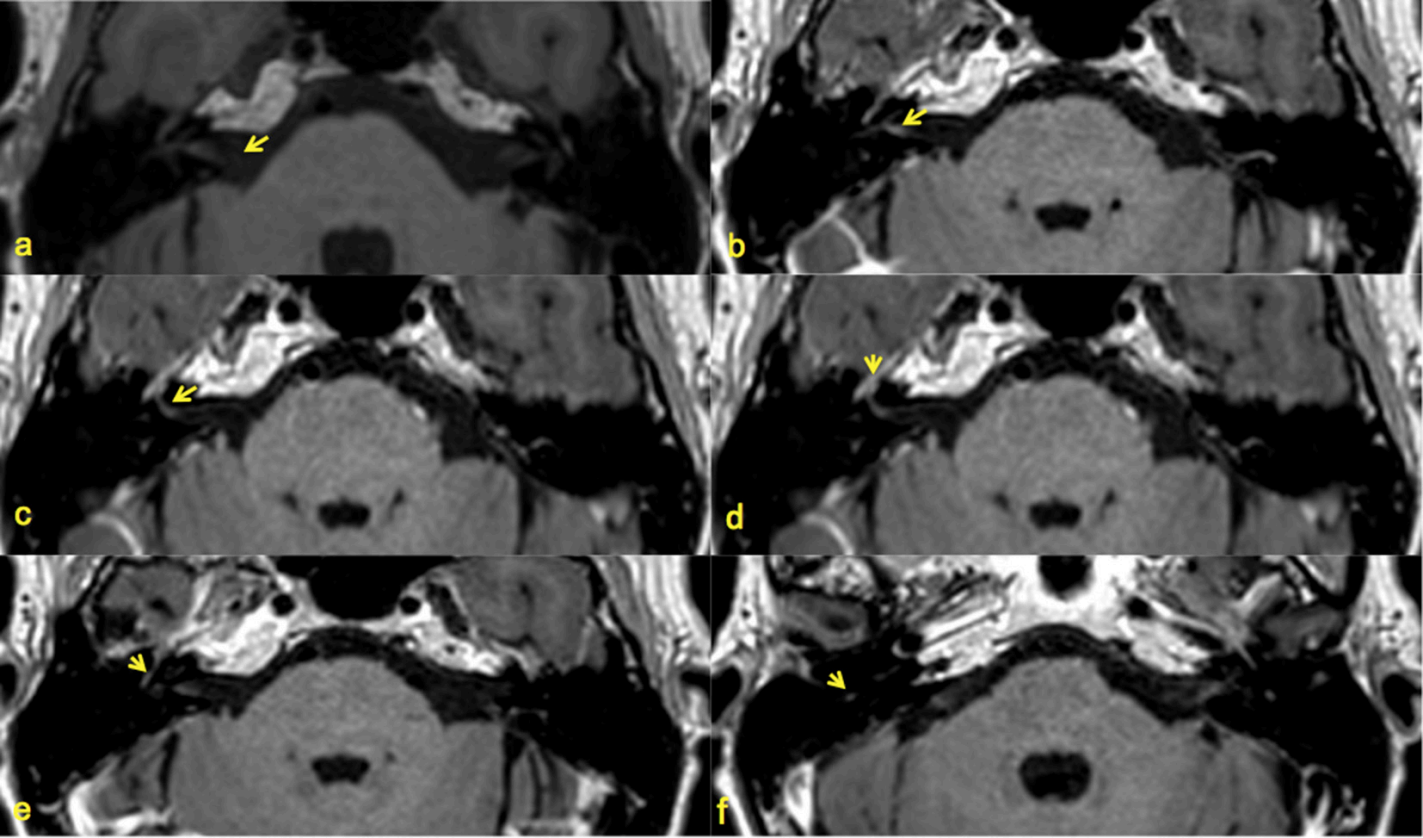
Individual facial nerve segments assessed. 3D T1W Fast Spin Echo (FSE) imaging at level of pons and internal auditory canals identifying (arrows) the facial nerve segments (a) Medial canalicular, (**b**) Fundal canalicular, (**c**) Labyrinthine, (**d**) Geniculate Ganglion, (**e**) Tympanic and f) Mastoid. Images magnified for display.

**Figure 2.**
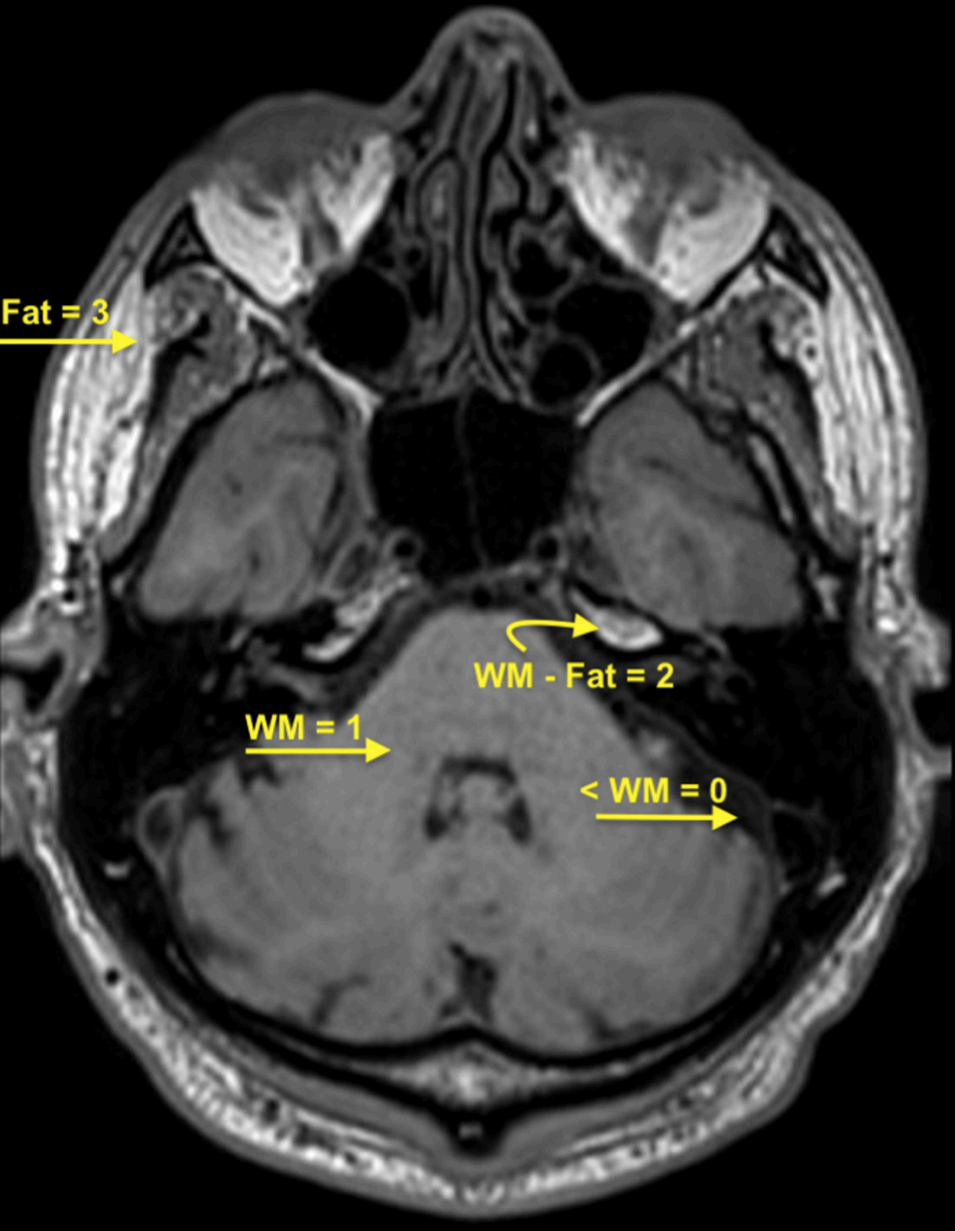
Facial nerve imaging intensity scoring system 3D T1W Fast Spin Echo (FSE) imaging at level of pons and internal auditory canals in a 38-year-old female. Images magnified for display. Signal intensity was assigned a value 0–3 (0, less than signal of the brain stem; 1, intensity of brain stem parenchyma; 2, signal intensity between brain stem and subcutaneous fat; 3, intensity of fat).

**Figure 3.**
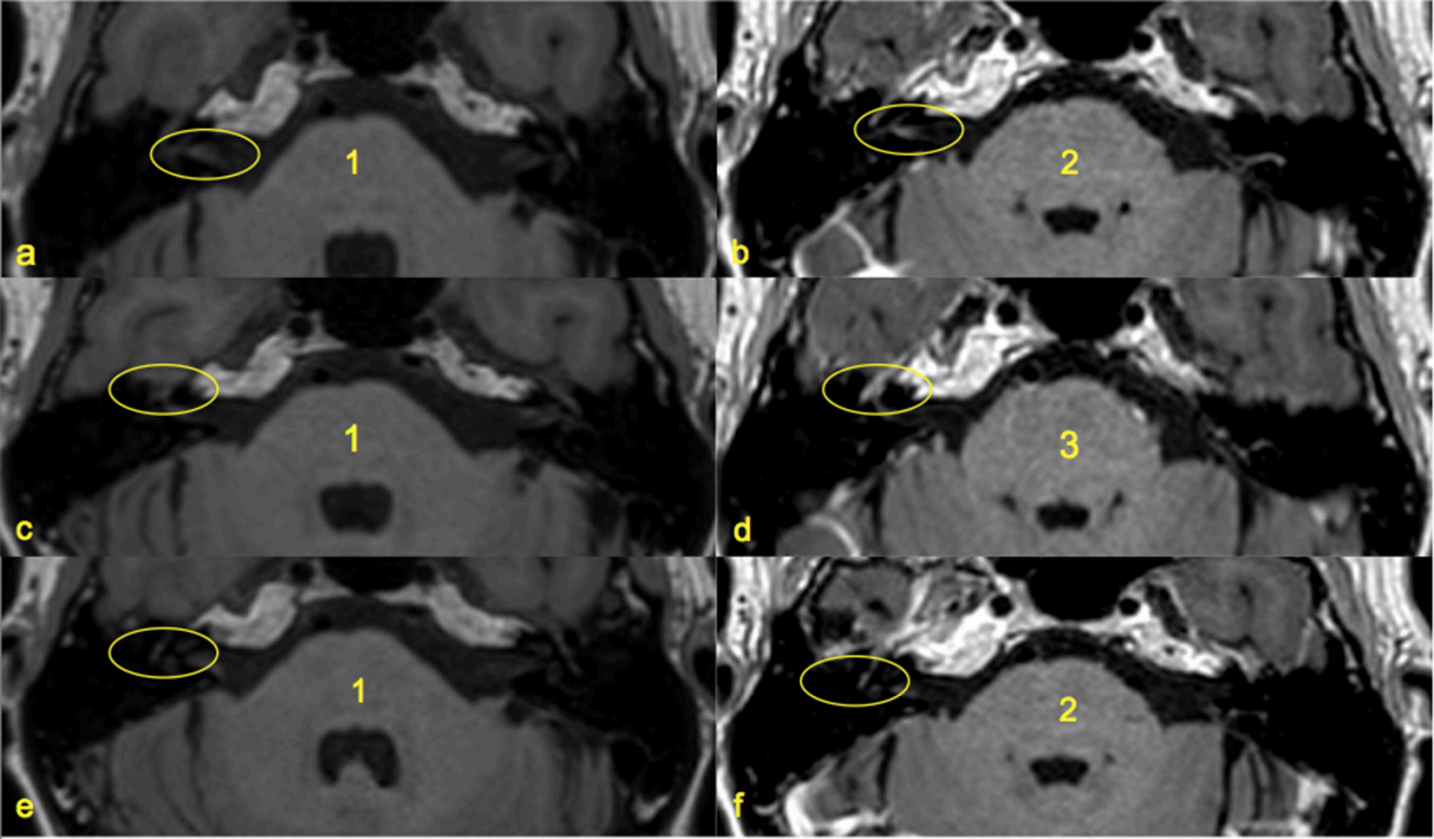
Examples of scoring of enhancement and grades of enhancement. 3D T1W Fast Spin Echo (FSE) imaging at level of pons and internal auditory canals identifying the scoring of facial nerve enhancement at: a) & b) Pre- and post-gadolinium at the fundal canalicular segment demonstrates an increase from a 1 to 2 score c) & d) Pre- and post-gadolinium at the geniculate ganglion demonstrates an increase from a 1 to 3 score e)& f) Pre- and post-gadolinium at the tympanic segment demonstrates an increase from a 1 to 2 score.

Two neuroradiologists (both with 9 years radiology experience) independently rated the facial nerves for signal intensity. The pre-gadolinium and post-gadolinium 3D T1W FSE sequences data sets were simultaneously reviewed for each patient. The individual facial nerve segments (medial canalicular, fundal canalicular, labyrinthine, geniculate ganglion, tympanic and mastoid) were scored (Figure 1). A Likert type scale was utilised with the signal intensity of the facial nerve segment scored as: 0, less than signal of the brain stem; 1, intensity of brain stem parenchyma; 2, signal intensity between brainstem and subcutaneous fat; and 3, intensity of subcutaneous fat (Figures 2 and 3). Final scores were achieved by consensus with a third neuroradiologist arbitrating for any disagreement.

### Statistical analysis

The descriptive data were collated per individual facial nerve segment, both pre- and post-gadolinium administration. The gadolinium enhancement grade was recorded as the difference in signal intensity score at each facial nerve segment between pre- and post-gadolinium 3D T1W FSE sequences. This was performed for CUBE and SPACE sequences, both individually and in combination. Cohen’s κ coeffcient was used to evaluate for interobserver agreement. The differences in enhancement grades were compared between the two ears as an indicator of any asymmetry. The differences in enhancement grades between the CUBE and SPACE sequences were also compared to determine the impact of using different proprietary sequences. Data was not normally distributed as analysed by the Kruskal-Wallis test. Wilcoxon signed-rank test was thus used for all statistical comparisons with *p* < 0.05 used to test for statistical significance.

## Results

There were 73 patients initially evaluated, however, nine patients met exclusion criteria resulting in 64 patients (21 male, 43 female; mean age 46, age range 24–85). This included 3D T1W SPACE (Siemens) (*n* = 43) or CUBE (GE) protocols (*n* = 21). A total of 128 facial nerves and 768 facial nerve segments were reviewed. There was substantial inter-observer reliability for the pre-gadolinium intensity with κ = 0.674 (95% CI: 0.508–0.841) and almost perfect inter-observer reliability for the post-gadolinium intensity with Kappa-=0.964 (95% CI: 0.945–0.984).

Initial disagreements between the observers for 26 out of 768 segments were resolved after open discussion.

All facial nerve segments demonstrated enhancement in some subjects and there was enhancement along at least one segment of the facial nerve in all subjects. Table 1 demonstrates the pre- and post-gadolinium signal intensity scores and Table 2 demonstrates the grade of enhancement at each segment for 3D CUBE and 3D SPACE sequences, both individually and in combination.

**Table 1.** Comparison of signal Intensity at each facial nerve segment for SPACE and CUBE 3D T1W FSE sequences alone and in combination

Segment	SPACE PRE	SPACE POST	*P* Value	CUBE PRE	CUBE POST	*P* Value	Combined Pre	Combined Post	*P* Value
**Medial canalicular**	1 (0.22)	1.02 (0.15)	0.16	1 (0)	1 (0)	1	1 (0.18)	1.02 (0.13)	0.2
**Fundal canalicular**	1.02 (0.15)	1.16 (0.4)	<0.05	1 (0)	1.19 (0.397)	<0.05	1.02 (0.13)	1.17 (0.4)	**<0.05**
**Labyrinthine**	1.02 (0.15)	1.02 (0.15)	0.32	1 (0)	1.02 (0.154)	0.32	1.02 (0.13)	1.02 (0.15)	0.2
**Geniculate Ganglion**	1.06 (0.24)	2.01 (0.33)	<0.05	1.02 (0.154)	2 (0.22)	<0.05	1.05 (0.21)	2 (0.29)	**<0.05**
**Tympanic**	1.05 (0.21)	1.38 (0.54)	<0.05	1 (0)	1.63 (0.48)	<0.05	1.03 (0.17)	1.47 (0.53)	**<0.05**
**Mastoid**	1.01 (0.11)	1.38 (0.51)	<0.05	1 (0)	1.38 (0.49)	<0.05	1 (0.09)	1.38 (0.50)	**<0.05**

Data reported as mean (SD) assigned signal intensity value from a visual inspection scale, whereby each facial nerve segment was assigned a value of 0–3 (0, less than signal of the brain stem; 1, white matter intensity of brain stem parenchyma; 2, signal intensity between brainstem white matter and subcutaneous fat and; 3, intensity of subcutaneous fat)

**Table 2.** Grades of enhancement of facial nerve segments SPACE and CUBE 3D T1W FSE sequences

Enhancement	SPACE 1 Grade of enhancement	SPACE 2 Grades of enhancement	SPACE Total grades of enhancement	CUBE 1 Grade of enhancement	CUBE 2 Grades of enhancement	CUBE Total grades of enhancement	OVERALL CUBE + SPACE grades of enhancement	*P* Value
**Medial canalicular**	2	0	2/86 (2%)	0	0	0/42 (0%)	2/128 (1.6%)	0.2
**Fundal canalicular**	12	1	13/86 (15%)	8	0	8/42 (19%)	21/128 (16%)	0.3
**Labyrinthine**	0	0	0/86 (0%)	1	0	1/42 (2%)	1/128 (1%)	0.1
**Geniculate Ganglion**	82	0	82/86 (95%)	41	0	41/42 (98%)	123/128 (96%)	0.3
**Tympanic**	31	0	31/86 (36%)	27	0	27/42 (64%)	58/128 (45%)	<0.05
**Mastoid**	32	0	32/86 (37%)	16	0	16/42 (38%)	48/128 (38%)	0.5

There was a statistically significant difference between the signal intensity on pre- and post-gadolinium 3D T1W sequences (*p* < 0.05) for fundal canalicular (16% enhancing), geniculate (96% enhancing), tympanic (45% enhancing) and mastoid (37% enhancing) segments (Table 1).

A similar pattern of gadolinium enhancement (*p* < 0.05) was exhibited at the different facial nerve segments by the SPACE and CUBE sequences (Table 2). CUBE only appeared to show significantly more tympanic segment enhancement than SPACE (64% v 36%, *p* < 0.05).

There was no statistical significant difference (*p* > 0.05) in the enhancement grades between the two petrous bones with only 12% nerves demonstrating an asymmetry in the degree of enhancement (0% medial canalicular, 8% fundal canalicular, 2% labyrinthine, 4% geniculate ganglion, 7% tympanic and 6% mastoid segments; some scores at sites were overlapped). There were 9/768 segments with a score three post-gadolinium signal intensity score.

## Discussion

Isotropic 3D T1W FSE techniques are now widely used in routine neuroimaging protocols. Proprietary sequences include CUBE (GE), SPACE (Siemens), VISTA (Volume Isotropic Turbo spin echo Acquisition) (Philips) and IsoFSE (Hitachi). They are all characterized by short non-spatially selective radio-frequency pulses to significantly shorten the echo spacing and variable flip angles for the refocusing radio-frequency pulses, which suppress blurring whilst reducing flow and chemical shift artifacts. They demonstrate a high signal-to-noise ratio and spatial resolution relative to spin echo sequences and they are less sensitive to flow related artifacts or susceptibility artefact compared to 3D GRE techniques.^7^ These 3D FSE techniques may be applied to T1W pre- and post-gadolinium imaging and the high spatial resolution isotropic data are optimal for imaging the facial nerve at the skull base. The isotropic data allow oblique reformatting along the course of the facial nerve and aids both comparison of the two sides or with previous MRI studies.^8^

Dedicated imaging investigation of the facial nerve is most frequently required in patients with a facial nerve palsy when there is an atypical cause for a Bell’s palsy; however, the facial nerve is often visualised within the skull base as part of routine brain imaging performed for a range of neurological presentations.^9^ Thus, an understanding of normal patterns of gadolinium enhancement of the facial nerve is a prerequisite to the interpretation of normal and pathological appearances in patients with and without facial nerve dysfunction. The range of normal gadolinium enhancement in healthy subjects has been reported in the context of T1W SE^1,2,5^ and more recently with 3D T1W GRE sequences;^3,4^ however, there has been no evaluation of the patterns when applying contemporary 3D T1W FSE techniques.

Normal facial nerve enhancement should be distinguished from that due to contrast leakage into the endo-neural space or venous congestion secondary to pathological processes.^4^ The physiological enhancement is thought to be due to the rich circum-neural arteriovenous plexus within the perineurium and epineurium of the nerve. Our study confirmed previous findings showing that the geniculate ganglion and tympanic segment demonstrate the most consistent and avid gadolinium enhancement,^1–6^ which is felt to correspond to the distribution of the perineural vascular plexus.^1,10^ Earlier studies using T1W SE 3 mm sections did not reveal enhancement at the fundus of the internal auditory meatus (IAM) and it was accepted to be a pathological appearance that was frequently demonstrated in the setting of a Bell’s palsy.^1,2,11^

More recent studies have focused on 3D T1W GRE sequences and these have shown increased facial nerve enhancement compared to T1W SE sequences^4,6^ with mild-to-moderate enhancement demonstrated at the fundus of the IAM in 3–15% of normal individuals.^3,4^ We demonstrated similar findings when applying contemporary 3D T1W FSE sequences, with fundal canalicular enhancement being present in 16% of cases. Although the presence of capillaries in the meningeal layers at the fundus of the IAM has long been recognised,^12,13^ there has been recent work demonstrating the existence of perineural pillars and villi associated with blood vessels in the internal auditory meatus.^14^ This is particularly important to recognise in normal subjects, since misinterpretation of physiological enhancement within the IAM may result in a false diagnosis of vestibular schwannoma, leptomeningeal disease or a Bell’s palsy. Correlation with high-resolution T2W sequences may aid the exclusion of a small intracanalicular schwannoma when such gadolinium enhancement is present.

The more conspicuous gadolinium enhancement using high-resolution isotropic spin echo relative to 3 mm slice thickness spin echo sequences is felt most likely to result from reduced partial volume effects and improved detection of the facial nerve signal. Other less likely explanations include differences in the timing of k space filling with respect to the gadolinium injection, and alterations in the degree of T1W due to the variable flip angle.

Although a similar pattern of gadolinium enhancement was exhibited on the SPACE and CUBE sequences, CUBE showed more tympanic segment enhancement than SPACE and only SPACE showed enhancement in the medial canalicular segment (2% of nerves). There was no significant asymmetry in the facial nerve enhancement at any segment. Asymmetry in the grade of enhancement was demonstrated in only 12% of cases, which is less than has been previously reported.^2^

We recognised the importance of evaluating the signal of the facial nerve prior to gadolinium in order to exclude apparent enhancement due to T1W shortening of the nerve using isotropic 3D spin echo techniques. Indeed, the variation in facial nerve scoring on pre-gadolinium sequences emphasizes the benefits of obtaining comparable pre-gadolinium sequences in neuroimaging protocols.

Our results may not necessarily be generalised to all 3D T1W FSE imaging scenarios. For instance, application on 3T MRI systems with higher signal-to-noise ratio (SNR) may influence conspicuity and signal returned by the facial nerve.^3^ Similarly, the use of post-gadolinium fat suppressed sequences may alter the dynamic range of contrasts and appreciation of signal intensity as the ambient contrast is changed.^15^ It could be speculated that differing physicochemical properties of gadolinium based contrast agents (GBCA) such as viscosity, osmolality and longitudinal relaxivity (r1) as well as injected volume may influence the physiological T1 shortening of the facial nerve and its perineural vascular plexus; however, a previous comparison of normal facial nerve enhancement with gadobutrol and gadopentate dimeglumine did not reveal any significant differences.^6^

There are some limitations of the study. Firstly our study population were not healthy volunteers, although strict exclusion criteria mitigated against the possibility of any facial nerve pathology. Secondly, our SPACE and CUBE studies were not matched and hence it is difficult to separate a difference in performance from physiological variation in enhancement between individuals undergoing the different proprietary sequences. Thirdly, our grading system was qualitative since we found the quantitation of signal intensity in the irregular narrow facial nerve to have unacceptable standard deviation. This may, however, limit its ability to detect lesser degrees of enhancement if the pre- and post-gadolinium facial nerves fall within the same signal intensity score definition. Finally, each facial nerve segment was analysed in a different plane; however, this applied to both pre- and post-gadolinium sequences so is felt unlikely to impact on the results.

## CONCLUSION

This is the first study to assess normal patterns of skull base facial nerve enhancement on contemporary 3D T1W FSE sequences. We emphasise that fundal canalicular enhancement should not necessarily be interpreted as pathological as has been suggested in paradigms derived from previous studies.
